# Supplementary material for: ComplexityMeasures.jl: Scalable software to unify and accelerate entropy and complexity timeseries analysis
Source: PLoS One. 2025 Jun 13;20(6):e0324431. doi: 10.1371/journal.pone.0324431 (PMC12165401; doi:10.1371/journal.pone.0324431)
Supplement: Attachment — Submitted filename: plos_one_review.pdf [file pone.0324431.s001.pdf]

Manuscript No. PONE-D-24-22899

ComplexityMeasures.jl: scalable software to unify and accelerate entropy and complexity timeseries analysis.

In this work the autors have introduce *ComplexityMeasures.jl.* It is an open-source software designed to efficiently implement a wide range of complexity and entropy measures for nonlinear time series analysis. They claimed that present software 1530 measures in just 3,834 lines of code. It indicates that, averages 2.5 lines per measure (version 3.5) due to its mathematically rigorous and composable design. This software offers superior computational performance, reliability, and extensibility compared to alternatives. As part of the *DynamicalSystems.jl* library, it supports sustainable development practices and provides researchers with a powerful tool for selecting appropriate measures and accelerating complexity-related research.

In conclusion, *ComplexityMeasures.jl* offers a scalable and high-performance solution for computing a wide range of complexity measures, outperforming alternatives by up to 1,000x and enabling practical applications previously limited by computational resources. Its composable design allows for easy creation of new measures, and its extensibility ensures instant usability across the broader ecosystem. Built as an open-source community project on GitHub, *ComplexityMeasures.jl* emphasizes openness, collaboration, and reproducibility, with over 200 pull requests and 140 closed issues since its inception. We encourage researchers to contribute new complexity measures directly to the software, benefiting both individual researchers by providing instant accessibility and recognition, and the wider community by enhancing reliability and testing within the *DynamicalSystems.jl* ecosystem. This approach not only improves the accuracy of new research but also promotes the openness of academic code, an important principle for scientific progress.

This should be immediately accept for publication.
