# Supplementary material for: ComplexityMeasures.jl: Scalable software to unify and accelerate entropy and complexity timeseries analysis
Source: PLoS One. 2025 Jun 13;20(6):e0324431. doi: 10.1371/journal.pone.0324431 (PMC12165401; doi:10.1371/journal.pone.0324431)
Supplement: Attachment — Submitted filename: response.pdf [file pone.0324431.s002.pdf]

Dear Editor(s)

Please find attached our responses below; they also outline the changes in the manuscript.

Reviewer #1:

- RESPONSE: We thank the reviewer for the overall positive evaluation!

Reviewer #2:

The paper presents a novel software solution ComplexityMeasures.jl for complexity measures in timeseries analysis. The novel software includes a vast number of measures, while the authors claim high computational performance, reliability, and extendability. The paper is interesting and it clearly presents a fine addition to the body of knowledge.

- RESPONSE: We thank the reviewer for the overall positive evaluation!

The Introduction is rather broad, and nicely presents the motivation and the rationale for complexity measures calculation showing its relevance. It starts with a discussion on entropy and its relation to complexity measures, giving an example of complexity measure and focusing on the problem of computability due to combinatorial explosion.

However, I would like to see Section 1.3. a bit more expanded. It emphasizes novelty of the paper with the innovative software design.

However, I think it should also emphasize 2-3 main contributions of the paper, bullet-by-bullet.

- RESPONSE: We thank the reviewer for the positive comments, however, we are really not sure what is requested here. The second and third paragraphs of section 1.3 already describe all the features of the software that should be highlighted in the paper. The paper by itself, without the software, does not have any "contributions" to highlight, as (on purpose) we haven't performed any novel research in this paper. We hope that the revisions done to address the other comments also address this one.

Also, some review of the other existing solution would be welcome in the introductory part, as well, not only in Section 4, where it is more focused on comparison.

- RESPONSE: We now refer to a list of existing software (alternative solutions) in the very first paragraph of the introduction. A lengthier discussion remains in Section 4.

Fig 1. is not really a figure, but merely a description of the prototype of the function. I think iy can be transformed into some kind of a diagram that can better depict the two central functions of the implemented ComplexityMeasures.jl. I think the composable structure of the solution should be presented graphically. Even some classification of complexity measures by type or elements that are needed in implementation in a form of a table would help to understand some design decisions. There are some discusions on it on lines 132-139.

- RESPONSE: We have updated the figure to be more illustrative of the composable design and have example inputs. In the figure caption we also refer to Table 2 that shows all possible inputs. Classifying complexity measures is not in the scope of our paper as we are not doing a literature review. Note that we have made the design decision (also stated explicitly in the paper) that every measure that is NOT a functional of a probability mass function or density is termed a ` ` complexity measure''. This may, or may not, align perfectly with individual publications using the same term.

Section 2 is missing information on the framework used to implement ComplexityMeasures.jl. Although I can assume it is written in Julia, I think the reader should not base his toughts on this kind of "assumptions", but should be explicitely given relevant details.

- RESPONSE: We now mention that ComplexityMeasures.jl is written for the Julia programming language in section 1.3.

Specifically, you should also discuss why you used Julia, and any elements of parallel programming if you used them in implementation.

- RESPONSE: We used Julia because it is performant, high level, it has overwhelmingly an open source community, and it has the best in class software for our ecosystem. We discuss this now in the start of the section 1.3 and cite some relevant resources.

In section 2.2, you state "To the best of our knowledge, there isn't any open source software in any programming language that provides such an extensive interface for extracting probabilities from data.". Can you back it up with some references to other APIs or frameworks, alternatives? Notably in Python and R?

- RESPONSE: The reviewer is correct to challenge the claim. To the best of our knowledge, we are not aware of a software having such an interface for probabilities even outside the context of complexity analysis. Of course, given the multitude of potential software that deal even remotely with statistics, checking everything is impossible. And we also don't have to, as we don't need to compete with every software remotely related to statistics. Therefore, we changed the statement to: "No other software on complexity analysis provides such an extensive interface for extracting probabilities from data." which we can say with certainty (and if someone does not believe us they can of course also check the long list of alternative software that we reference). We make the claim quantitative as well in Table 1. The row on outcome spaces shows that we are the only software that even defines the concept and has more than 1 estimator. Table 1 also compares with a Python software. We admit that the table does not explicitly compare versus an R software, but we compare we three of the most well-known/cited examples in the literature, and as such we believe we already provide a convincing case.

In several places you point out to the documentation of ComplexityMeasures.jl. Provide a link, and put it in the list of references.

- RESPONSE: Done.

Section 2.4. Provide a link to github project and reference it here.

- RESPONSE: Done.

Section 2.6. is too short. I think that the authors should provide the readership with implementation details of their software solution. As a researcher who is very interested and involved in parallelization, performance/code/memory optimizations and generally algorithm implementation, I disagree that "it is out of scope of the paper" to present such details in a paper which focuses on the software itself. I think each of those elements should be given its own paragraph or subsection, as it constitutes an important part of the solution. Both on the side of the choice of algorithms, paralellization techniques, data decomposition, and similar. If you claim that something is 1000x times faster than something else, you should back it up with reasons.

- RESPONSE: Rather than using reasons, it is even better for us to back up our claims with evidence. We provide ample evidence by providing the actual benchmark code we run in a public repository, and cite it in the table caption. As all of our code (and the Julia language) are open source, everything can be run by anyone for free and confirm our claims in case of doubt. This is

evidence enough regarding performance. For the rest of the comment, while we think it is great that the reviewer is interested in performance optimizations, we remain clear that such details are not the main focus of the paper and will not be the interest of the majority of our target audience which are users of the software. Besides, it would be impossible for us to track every single optimization we performed over the course of three years development and 140 pull requests. Nevertheless, we added an extra brief paragraph on parallelization (and why almost nothing in ComplexityMeasures.jl is parallelized) in section 2.6 that we hope will be enough to interest the reviewer to perhaps even have a look at the source code of the software!

Section 4, bullet 5. You should provide HW/SW testing environment and a more clear description on how you tested and compared your solutions to alternatives. Again, 10-1000x requires good explanations.

- RESPONSE: in the table description we added both where to find the codebase that runs the benchmarks, as well specs for the laptop that they were run on. Originally this information was only in the reproducibility subsection, but it is now clear to us that this information also should have been in the table caption, so we apologise for this.

Besides the response to the reviewer #2, we made a couple more changes for the revised version:

- Renamed CausalityTools.jl to Associations.jl, since this software changed its name since submission.

- Added FluctuationComplexity information measure to table 2, which has been added since submission.

- Updated the number of information definitions in table 1 from 11 to 12 (added the FluctuationComplexity information measure).

- Appendix S2: updated the total number of computable measures to account for the fluctuation complexity and other combinations.

- We replaced the "Multiscale forms" row with the "Multiscale functions" in table 1, which now states the total number of multiscale variants per package.

- We now refer to version 3.7 of ComplexityMeasures.jl, not version 3.5, as in the previous version of the manuscript.

- We updated the "Total entropy and complexity measures" row for EntropyHub to 26, after inquiring with the author of the package for updated counts.
